# Description of the African Cigarette Prices Project Data

**DOI:** 10.1016/j.dib.2024.110434

**Published:** 2024-04-18

**Authors:** Kirsten van der Zee, Senzo Mthembu

**Affiliations:** Research Unit on the Economics of Excisable Products, School of Economics, University of Cape Town, Cape Town, South Africa

**Keywords:** Tobacco, Cigarettes, E-cigarettes, Prices, Cigarette brands, Retail survey, Spaza shops, Street vendors

## Abstract

The African Cigarette Price Project is a project that collects tobacco prices from African countries. Amongst other things, the data enable users to estimate price differences across brands, urban/rural divides, types of packaging, retail types, and trends in price over time. A total of 215 354 individual prices were collected during the first twelve rounds of the project (collected biannually from 2016 to 2022). Data collection continues to date. Data have been collected from 19 African countries, with most data from South Africa, Zimbabwe, Lesotho, Namibia and Botswana. Other countries include Ethiopia, Malawi, Tanzania, Chad, Eswatini, Mozambique, Nigeria, Zambia, Ghana, Madagascar, Kenya, Mauritius, Uganda and Cameroon. The project employs a novel data collection approach, by contracting local and international University of Cape Town (UCT) students as fieldworkers to collect price data while at home over the long university vacation. The data were collected at the retail level; the lowest level of geographic detail available in the public use dataset is the suburb. While the price data are not nationally representative, the data collection method is simple and affordable and provides an indication of the range of prices and the brands available in the respective countries. While cigarette prices make up the bulk of the data, other common tobacco products included are hookah tobacco, snuff, pipe tobacco, cigars, e-cigarettes, hand-rolled tobacco, and others. The collection of these other tobacco products started in round 4 (2017).

Specifications Table

**Table:** 

Subject	Public Health and Health Policy.
Specific subject area	Tobacco control
Data format	Raw
Type of data	Stata, CSV
Data collection	The Research Unit on the Economics of Excisable Products (REEP), based in South Africa, recruits and trains registered University of Cape Town (UCT) students to be fieldworkers. The recruitment process occurs before the major university holidays (December/January and June/July). REEP recruits local (South African) and international African UCT students who spend their vacations in their hometowns or home countries. Fieldworkers must be locals in the countries/areas in which they conduct data collection. Fieldworkers conduct a random walk through their location and visit stores at random (as many as they choose). Stores visited are placed into one of three categories: retail outlets (formal stores), spaza shops (micro-convenience stores, typically in permanent structures) and street vendors (small, informal stalls, with non-permanent structures). Fieldworkers use their discretion to determine where to collect data, and the proportion of each outlet type they visit. The fieldworker asks the owner or manager of the store for permission to collect prices and take photos in the store. The fieldworker records each price in an Excel template provided to them by (REEP). The Excel template includes columns for the product type, price per unit, quantity per unit, name of the brand and sub-brand, outlet type, outlet name, currency, and date of collection. Each price must be verified with a photograph, as well as a photograph of the storefront. Fieldworkers are paid per price collected.
Data source location	The data have been collected from 19 African countries, namely Botswana, Cameroon, Chad, Ethiopia, Ghana, Kenya, Lesotho, Madagascar, Malawi, Mauritius, Mozambique, Namibia, Nigeria, South Africa, Eswatini, Tanzania, Uganda, Zambia and Zimbabwe.
Data accessibility	Repository name: DataFirst DOI: https://doi.org/10.25828/nvz2-ah77 URL: https://www.datafirst.uct.ac.za/dataportal/index.php/catalog/927/study-description The data are open access [1]. Users must create a free profile on the DataFirst website to download the data. Photos are not publicly available, but users can contact the project administrators to request specific photos if necessary (with motivation).
Related research article	[2] Z. Sheikh, R. Branston, K. van der Zee, A. Gilmore, How has the tobacco industry passed tax changes through to consumers in 12 Sub-Saharan African countries? Tob Control (2023). https://tobaccocontrol.bmj.com/content/early/2023/08/02/tc-2023–058054

## 1 Value of the Data

• The data provide tobacco control researchers and policymakers with an independent source of tobacco price data from African countries, where data is typically scarce. The data can be used to assess price trends in the region, such as price differences across brands, locations, types of outlets, packaging, and over time.
• The data can be used to investigate tobacco market dynamics, such as single stick sales, the presence of products with illicit features or tax shifting behavior (by the tobacco industry). It is important to understand these local market dynamics when formulating effective tobacco control policies.
• The primary beneficiaries or users of this data are researchers, policymakers and other stakeholders (for example, advocacy groups) who are interested in tobacco control and tobacco use, and who are specifically interested in changes in the retail price as a tobacco control tool (specifically, tobacco taxation).
• The data can be used to conduct univariate, bivariate or multivariate analysis. Users can conduct price comparisons by product features, location, and over time.
• The data includes prices for next-generation nicotine products (e.g. e-cigarettes). While these make up a small share of the data, the data include prices for various components of these products, including devices, cartridges, e-liquids, etc. This data can provide insight into e-cigarette availability, variability, and price in the region, and by extension, tobacco industry e-cigarette strategy.

## 2 Data Description

To date, the dataset consists of 12 rounds of data (cross-sections). The 13th round is currently being processed and cleaned to be made available in the coming months, and the 14th round is being collected. Of the publicly available data (12 rounds), 215 354 prices are included. These are available in a single, standardized file, in both DTA and CSV formats. Other files available for download are the fieldworker information pack (instructions for fieldworkers), and the letter from REEP, verifying that the student is a fieldworker for the project. Fieldworkers can present this letter (which is signed, stamped and includes the contact details of the project principal investigator) to store managers/owners.

**Fig. 1:**
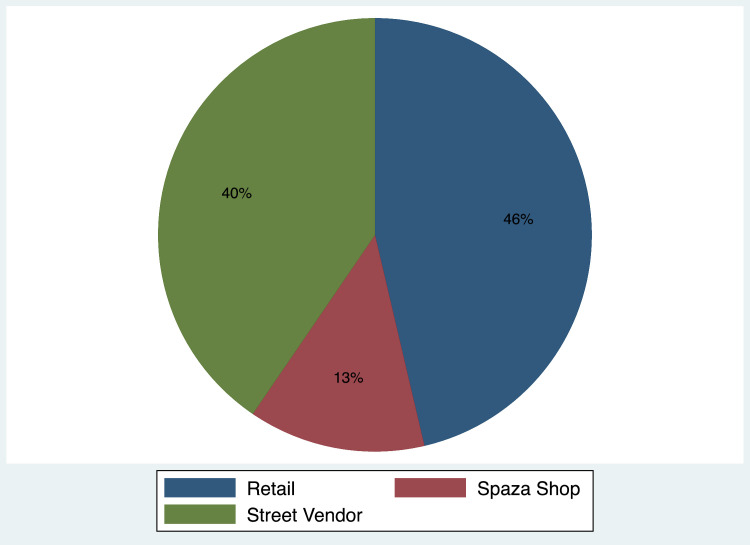
Share of each outlet type—African Cigarette Prices data, rounds 1 - 12.

Source: ACP Version 1.6

Table 1 below provides a description of the publicly available data, Table 2 provides the distribution of the data by country, and Fig. 1 depicts the distribution of the data by outlet type. Over 200 fieldworkers have been employed by the project, covering 19 African countries. Some fieldworkers have collected data during multiple rounds of the project. Data are collected from formal urban, informal urban, and rural areas. Fieldworkers visit formal tobacco-selling retail outlets, spaza shops (informal convenience stores), and street vendors in their home countries. Examples of the three store types are included in the section below.

**Table 1:** Description of the African Cigarette Prices data, rounds 1 - 12.

Round	Collection dates	No. prices collected	No. fieldworkers	No. countries	Products included
1	14 Jan 2016 –28 Jan 2016	1025	6	5	Cigarettes
2	2 Jun 2016 – 17 Jul 2016	9301	28	7	Cigarettes and Cigars
3	2 Dec 2016 – 28 Feb 2017	7 701	8	4	Cigarettes
4	12 Jul 2017 – 4 Sept 2017	10 901	23	7	All tobacco excl. ENDS
5	4 Dec 2017 – 18 Feb 2018	22 185	38	8	All tobacco excl. ENDS
6	12 Jun 2018 – 5 Aug 2018	23,140	34	8	All tobacco excl. ENDS
7	16 Nov 2018 – 1 Feb 2019	18 770	36	10	All tobacco incl. ENDS
8	4 Jun 2019 – 9 Aug 2019	23 982	30	10	All tobacco incl. ENDS
9	2 Nov 2019 – 16 Feb 2020	39 139	65	13	All tobacco incl. ENDS
10	17 Jun 2021 – 17 Sept 2021	24 352	40	10	All tobacco incl. ENDS
11	6 Dec 2021 – 11 March 2022	19 595	20	5	All tobacco incl. ENDS
12	16 June 2022 – 16 August 2022	15 263	16	4	All tobacco incl. ENDS

Source: ACP Version 1.6.

ENDS: Electronic Nicotine Delivery Systems.

**Table 2:** Number of observations per country—African Cigarette Prices data, rounds 1 - 12.

Country	Prices collected
South Africa	73,715
Zimbabwe	44,085
Lesotho	33,884
Namibia	32,217
Botswana	13,008
Ethiopia	6470
Malawi	2491
Tanzania	2441
Chad	1766
Eswatini	1302
Mozambique	956
Nigeria	760
Zambia	591
Ghana	485
Madagascar	367
Kenya	349
Mauritius	260
Uganda	141
Cameroon	66
**Total**	**215,354**

Source: ACP Version 1.6.

## 3 Experimental Design, Materials and Methods

### 3.1 Fieldworker recruitment

REEP recruits fieldworkers from amongst registered UCT students. To qualify as a fieldworker, a student must be a local^1^ from a qualifying location and be visiting this location during the vacation. Qualifying areas include any African country besides South Africa, as well as rural and township areas in South Africa.^2^ The fieldworker must have access to a smartphone with camera and have access to and be proficient in Microsoft Excel. Fieldworkers who are in the same cities/areas are required to coordinate their shop visits to avoid collecting data from the same stores, and the fieldworker coordinator oversees this process.

### 3.2 Data collection

Fieldworkers conduct a random walk of the areas in which they collect data, and choose how many and which stores to visit. Each fieldworkers cannot collect more than 1500 prices, unless special permission is obtained (for example, there are only a few fieldworkers in the area, and little or no data has been collected in the area before). This keeps the data review and management process manageable.

REEP provides fieldworkers with an Excel template to input the price information. The Excel template includes columns for the following: fieldworker code (supplied by REEP), country, currency, date of collection, location (province/state, city/town/village, suburb, and GPS coordinates), outlet type, retail outlet sub-type,^3^ name of outlet (in applicable), product type, brand, sub-brand, quantity, price per unit (local currency), storefront photo code, product photo code. There is also a column for fieldworker comments. The fieldworker does not use a formal questionnaire but takes note of all the information required to complete the template.

**Fig. 2:**
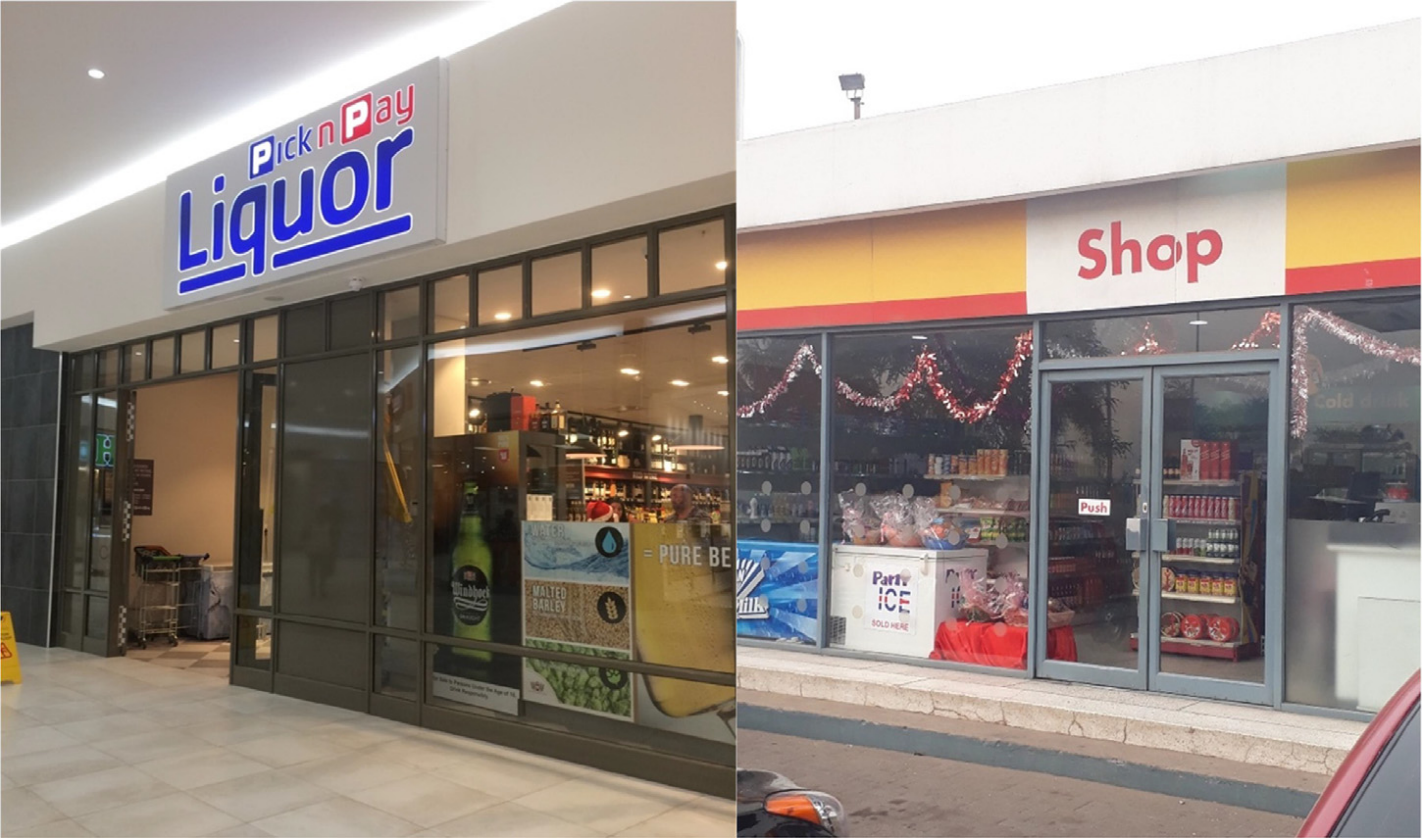
Examples of retail outlets (African Cigarette Prices data).

**Fig. 3:**
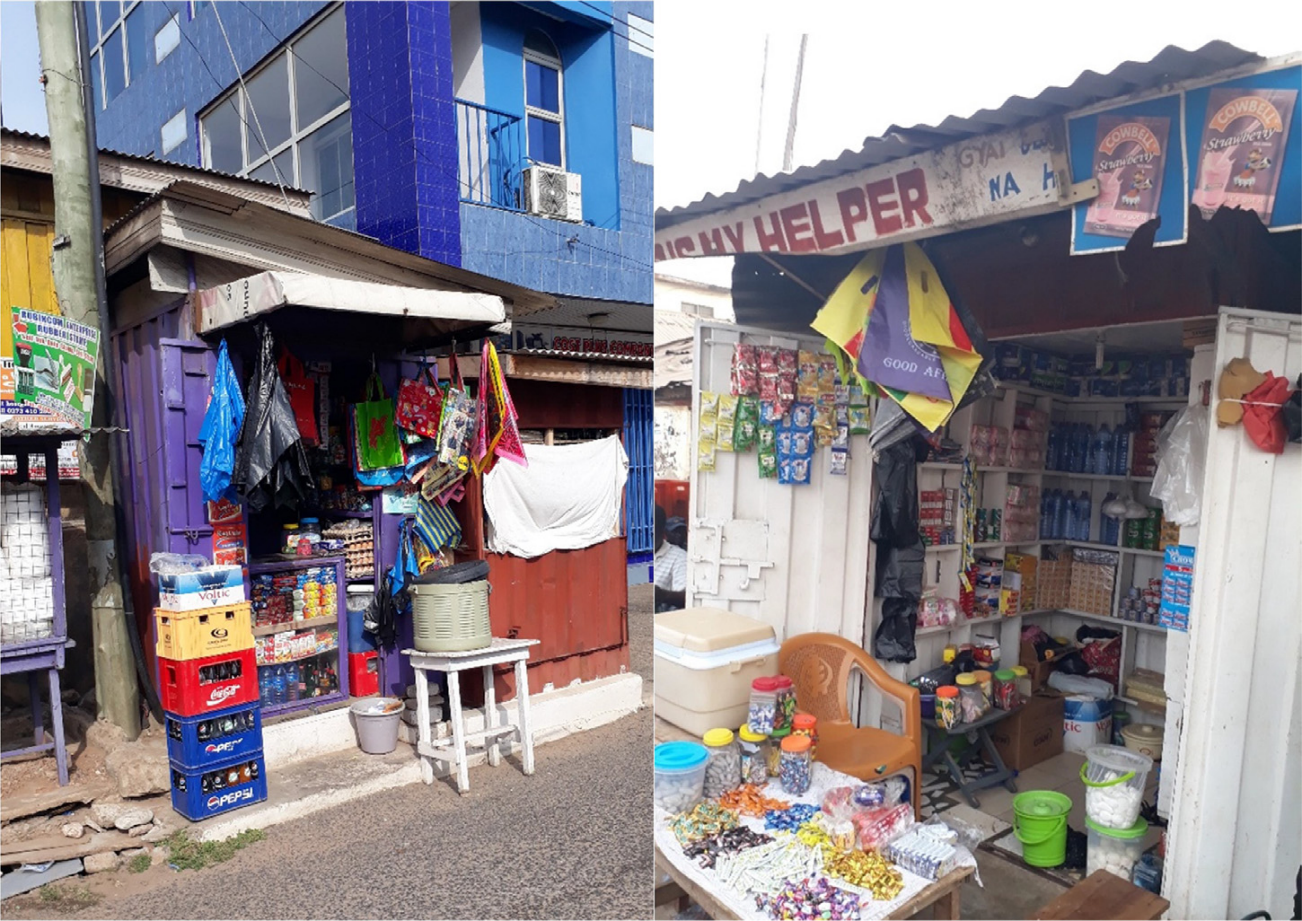
Examples of spaza shops (African Cigarette Prices data).

**Fig. 4:**
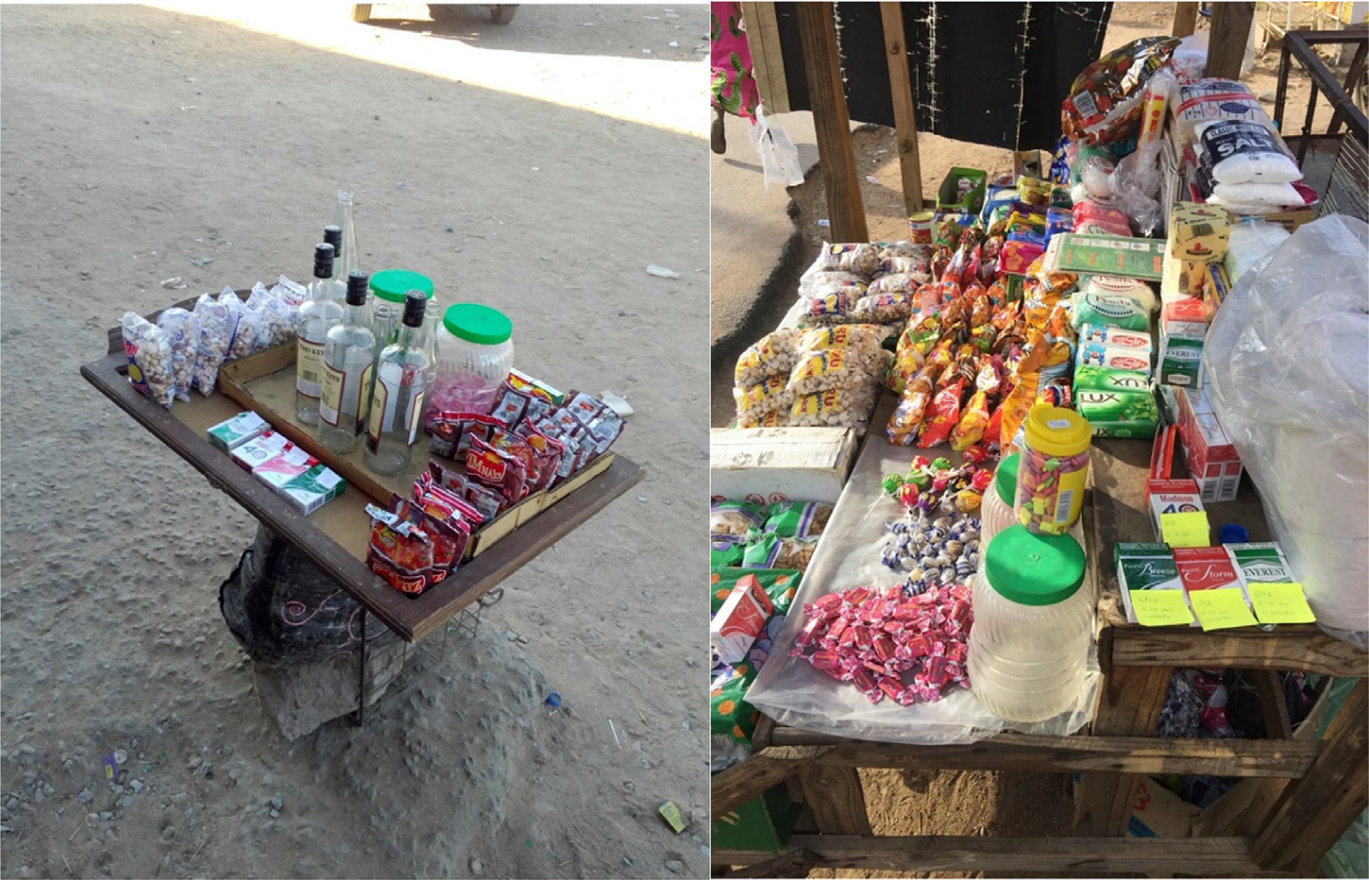
Examples of street vendors (African Cigarette Prices data).

For the outlet type, fieldworkers select either retail outlet, spaza shop or street vendor. Retail outlets are formal outlets, for example grocery stores or wholesalers. Spaza shops are semi-formal micro-convenience stores, typically with a range of grocery items, and located in permanent structures. Street vendors are informal stands, operating from non-permanent structures, and typically with a very small selection of goods (often only a handful). For the first four rounds of the project, fieldworkers could choose between only formal outlets and street vendors; however, given feedback from fieldworkers, in round 5, a third option, spaza shop, was added to the outlet categorization. Fig. 2, Fig. 3, Fig. 4 provide examples of the three outlet types.

**Fig. 5:**
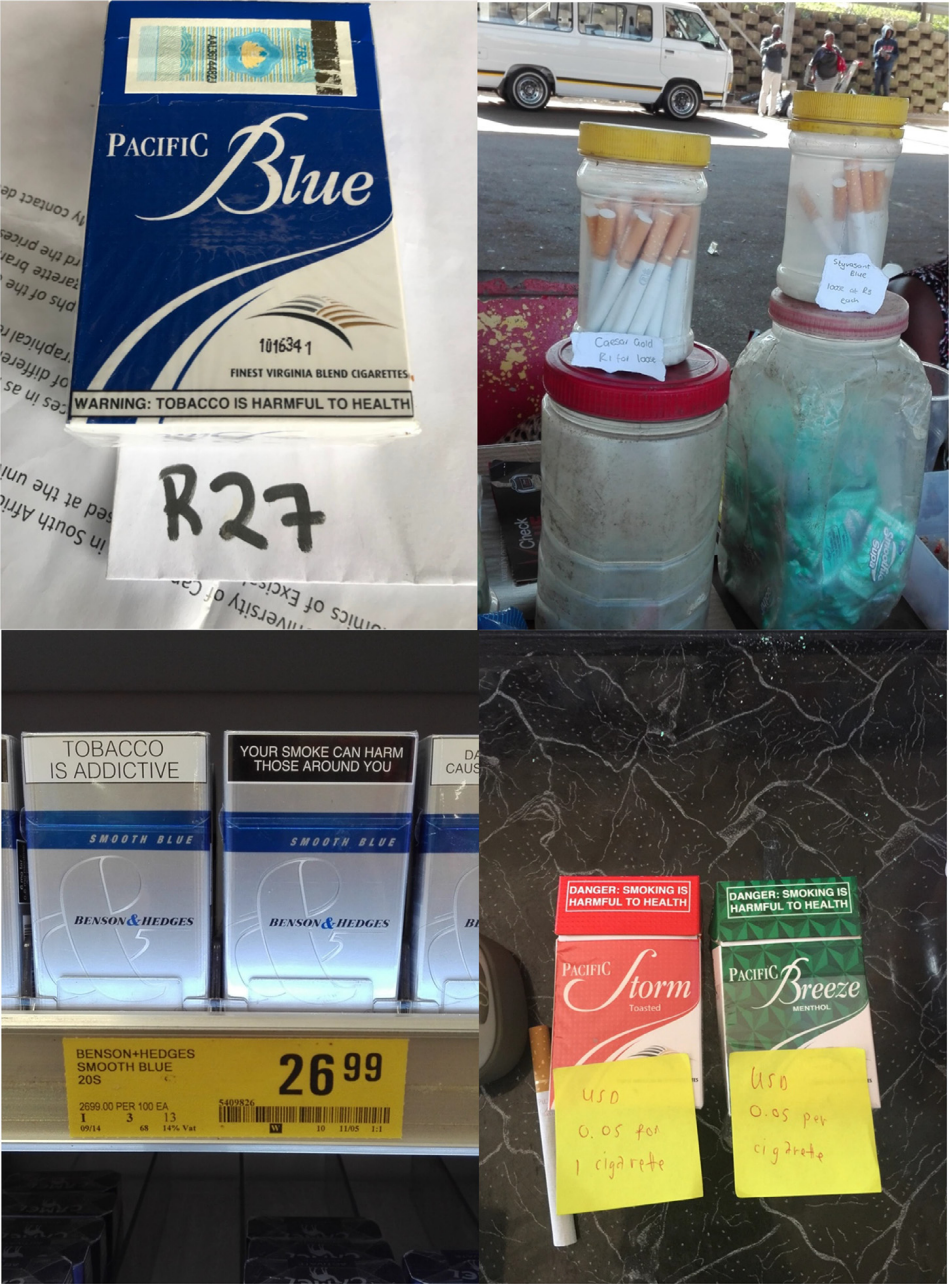
Examples of product photos (African Cigarette Prices data).

Fieldworkers must request permission from shopkeepers/vendors to record prices and take photographs of tobacco products. For each price collected, the fieldworker must take a photograph of the product, clearly showing the price, quantity, and branding. In cases where prices are not displayed (typically at street vendors and some spaza shops), they must write down the quoted price on a note and include this note in the photo. For each store, the fieldworker must also capture a storefront photograph; in the case of street vendors, they must capture the vendor's stand, without capturing the vendor him/herself (the above pictures of retail outlets, spaza shops and street vendors are examples of storefront photos). Fig. 5 provides examples of products photos, both from formal and informal outlet types.

The photographs are not released with the public dataset. The primary purpose of the photographs is for project administrators to validate that the data reflected in Excel are genuine. The photos are also often used to validate anomalies or identify mistakes in the Excel data.

After collecting the data, fieldworkers input the information relating to each price (such as the brand and sub-brand, store name, date, and address) into the Excel spreadsheet. Fieldworkers must store all photographs in a designated folder system on Dropbox. Fieldworkers name all the photographs according to a standardized naming system provided by the fieldworker coordinator; this allows each photo to be easily referenced along with its entry in Excel, and then identified in the photo folder.

### 3.3 Payment scale

Fieldworkers are paid per price collected, and at different rates depending on the store type. Prices from street vendors are remunerated at twice the rate as prices from retail outlets (spaza shop prices are renumerated at a rate mid-way between the rates for retail outlets and street vendors). This is because retail outlets typically have a very large range of products available, for example, a fieldworker might collect 10 times as many prices from a single retailer as from a single street vendor. Retailers are also typically well organised, so it is quicker and easier to collect many data points from them. Street vendors, and to a lesser degree spaza shops, typically have a much smaller selection of products (sometimes only one or two options) and generally do not use price tags, requiring fieldworkers to display the prices themselves. Applying a higher payment rate for street vendors and spaza shops incentivises fieldworkers to collect prices from these outlet types too. There is no prescription that fieldworkers should visit various types of stores, although we do encourage them to do so, to provide more variability and coverage in the data.

### 3.4 Post-data collection processing

After submission, the fieldworker coordinator (sometimes with the assistance of a team of data reviewers, depending on the number of fieldworkers) reviews and validates a random selection of each fieldworker's data, using the photographs submitted. In this step, the reviewers also check that the data are correctly inputted and are of a high quality. At this stage, the fieldworker may be required to make corrections to their data. Once each fieldworker's data has been reviewed, fieldworkers are paid per price. The data are then combined and uploaded to the open data portal, Datafirst.

### 3.5 Variables

There are 28 variables in the final dataset (Table 3). The twelve rounds have been appended, resulting in a final dataset with 215 354 observations. Since this is an ongoing project, an updated dataset is uploaded with each additional round collected.

**Table 3:** List of variables—African Cigarette Prices data, rounds 1 - 12.

Variable Name	Label (description)
Store_ID	Unique store ID
Round†	Round of data collection (1–9)
Fieldworker Code	Fieldworker code (ID)
Country	Country
Currency	Currency
Day	Day
Month	Month
Year	Year
Date	Date
Province	Province (or state if applicable)
City	City (or town or village)
Suburb	Surburb
Outlet_Type	Outlet type - retailer, spaza shop* or street vendor
Retail_Subtype	Retail outlet subtypes (a description of the type of outlet if formal, i.e. grocery store, petrol station, tobacco specialist store, airport, etc.)
Outlet_Name	Name of outlet
Product	Type of tobacco product
Brand	Name of brand
Sub_Brand	Sub-Brand
Quantity	Number of cigarettes in pack (1 if single)
Unusual_Quantity_Flg†	Indicator for if the quantity of cigarettes is non-standard (e.g. 3 sticks per pack)⁎⁎
Local_Price	Price in local currency (as on price tag)
Local_Price_Per_Stick_Cigarette†	Local price per stick (per cigarette)
Dollar_Exchange_Rate†	Dollar exchange rate for the relevant period
Dollar_Price†	Dollar price for this pack
Dollar_Price_Per_Stick_Cigarette†	Dollar price per stick (Cigarettes)
Dollar_Price_Pack_Cigarettes†	Dollar price per standard pack of 20 (=price per cig*20)
Fieldworker_Comment	Fieldwork comments
Data_Cleaner_Comment†	Data Cleaner Comment

⁎ Spaza shops are informal convenience shops.

⁎⁎ Standard cigarette packaging in the region is single (loose) stick, 10 pack, 20 pack, 30 pack or carton of 200. Occasionally packs of 2 or 3 are available. Any prices for quantities other than these are flagged and investigated, to ensure that fieldworkers are not manipulating the data to generate more prices. Collecting prices outside of the standard packaging units is discouraged unless it is a genuine product offering.

† Variables derived post-data collection.

## Limitations

The data are not representative at any geographical unit. This is because fieldworkers use their discretion in deciding where to collect data (within the bounds outlined by the fieldwork coordinator, for example, a specific town), and what share of data to collect from each outlet type.

Since fieldworkers are paid per price collected there is an incentive for them to falsify data. Fieldworkers are required to submit photographs as proof for each price. These photographs are used to validate a random sample of each fieldworker's data. Every effort is made to ensure that the data uploaded to DataFirst is genuine.

## Ethics Statement

The project has been granted ethics clearance from the University of Cape Town through the Faculty of Commerce. The ethics clearance number for the project is REC2021/05/011. The data collection does not directly involve human subjects, beyond requesting permission to take photographs in the stores. Every effort is made to avoid including any people in the photos, in any identifiable way. Furthermore, photos are not made publicly available. Sensitive information (e.g., GPS location) is removed before the final data are published.

## CRediT authorship contribution statement

**Kirsten van der Zee:** Conceptualization, Writing – review & editing, Investigation, Validation, Visualization. **Senzo Mthembu:** Methodology, Data curation, Writing – original draft, Visualization.

## Data Availability

African Cigarette Prices 2016-2022 (Original data) (Data first).

## Appendix Supplementary materials

Image, application 1

Image, application 2

## Footnotes

1 Fieldworkers must be locals from their data collection area (preferably speaking the local language), so that they collect the most accurate prices. This is most important when collecting data from informal traders who may not display price labels, when non-locals may attract higher (“tourist”) prices.

2 Statistics South Africa (StatsSA) regularly collects cigarette price data in South Africa. However, these data are not publicly available, and StatsSA does not comprehensively collect prices in informal areas. For this reason, ACP collects data in rural/township areas in South Africa, and from informal/street vendors in some urban areas.

3 For retail outlets, fieldworkers must provide more detail about the type of retail outlet, for example grocery store versus gas station versus wholesaler. This is because prices can vary significantly between these types of outlets.
